# Aging reprograms the functional, epigenetic, and metabolic landscape of macrophages

**DOI:** 10.21203/rs.3.rs-10155437/v1

**Published:** 2026-06-30

**Authors:** Manasa Suresh, Francisco Tapia Belmonte, Bryan T. Weselman, Xintang Li, Marie Durr, Isabella Duchovny, Yanitza Gutierrez-Monsalve, Matias I. Hepp, Satish Kumar R. Noonepalle, Alexis Salas-Burgos, Alejandro Villagra

**Affiliations:** Georgetown University, Lombardi Comprehensive Cancer Center; Concepcion University; Georgetown University, Lombardi Comprehensive Cancer Center; Georgetown University, Lombardi Comprehensive Cancer Center,; Georgetown University, Lombardi Comprehensive Cancer Center,; Georgetown University, Lombardi Comprehensive Cancer Center; Universidad Católica de la Santisima Concepción; Universidad Católica de la Santisima Concepción; Georgetown University, Lombardi Comprehensive Cancer Center; Concepcion University; Georgetown University, Lombardi Comprehensive Cancer Center

**Keywords:** Macrophages, Immuno-senescence, Epigenetics, Metabolism

## Abstract

Immuno-senescence is a dominant risk factor of chronic diseases, yet the impact of aging on macrophages remain understudied, despite their involvement in age-related conditions. Here, we define macrophage aging by integrating transcriptomic, epigenetic, metabolic, and functional analyses across the lifespan. Besides aging hallmarks, we uncovered progressive changes in macrophages, including reduced responsiveness to both inflammatory and anti-inflammatory cues and an imbalanced cytokine and chemokine secretory profile. This age-driven remodeling is supported by significant rewiring of epigenetic regulators, particularly histone-modifying genes, alongside coordinated shifts in metabolism toward lipid activation and trafficking at the expense of mitochondrial redox programs. Strikingly, these alterations differ between M1- and M2-like macrophages, indicating phenotype-specific aging trajectories. Functionally, aged macrophages exhibit enhanced phagocytic capacity, diminished migratory ability, and preserved antigen presentation. Together, our findings establish that aging does not simply impair macrophages; it drives an active, multi-layered reprogramming process, providing a framework to address inflammaging and age-associated diseases.

## INTRODUCTION

Macrophages are specialized innate immune cells that are either tissue-resident (such as Kupffer cells, microglia, alveolar macrophages, etc.) or derived from circulating bone marrow-derived monocytes^1^. They play a crucial role in innate immune regulation and surveillance and influence the effector adaptive immune responses during chronic diseases^2–5^. Their key functions are phagocytosis, antigen presentation, chemotaxis-driven migration, wound healing, and cytokine secretion^4,6^. Macrophage phenotype and function are mainly dictated by cytokine and chemokine signals from the surrounding microenvironment^7^. Traditionally, they are classified into two very broad categories: pro-inflammatory M1-like macrophages, which drive immune activation and antimicrobial responses, and anti-inflammatory M2-like macrophages, which promote tissue repair and immune regulation. However, this binary classification is oversimplistic, as macrophages exhibit phenotypic plasticity, often in hybrid states between these two extremes. Given their diverse functions in health and disease, there is growing interest in targeting macrophages or harnessing their therapeutic potential as tools to fight cancer, chronic wounds, and autoimmune disorders^4,8–12^.

Aging is a natural process with established hallmarks including genetic instability, telomere attrition, and epigenetic alterations that contribute to decline in cellular functions and increase susceptibility to chronic diseases^13^. Studies across various species have demonstrated age-related epigenetic alterations including histone modifications, DNA methylation, chromatin remodeling, non-coding RNA regulation, and RNA processing that play a crucial role in regulating gene expression^14–16^. Senescence-associated secretory phenotype (SASP) has been described to promote inflammation with aging, commonly termed as “inflammaging”^17^. While there is still no consensus on all the factors contributing to SASP, known members include cytokines, chemokines, inflammatory and growth factors, and non-protein soluble factors^18,19^. The phenomenon of inflammaging can also describe the decline in cellular repair and the increase in genomic damage with aging, linked to low-grade inflammation, which has been associated with macrophage function^20^.

The immune system efficacy gradually declines with aging, commonly known as immuno-senescence^21^. Age-related immune remodeling has been extensively described for T-cells and, to a lesser extent, for B-cells, including changes in subtypes, decreased effector functions, dysregulated antigen responses, and altered metabolic activity^22,23^. The impact of aging on myeloid cells is not well reported, and despite the growth in our understanding of macrophage biology over the past decade, the implications of aging on their functions remain incompletely understood. Given their undeniable role in many age-related conditions, such as diabetes, cancer, atherosclerosis, cardiovascular diseases, chronic inflammatory diseases, and Alzheimer’s disease, it is significant to understand the changes in aging macrophages. For example, epigenetic changes, specifically histone modifications, have been observed in healthy aging^24^; however, their role in macrophage aging and the impact on phenotype and functions remain unexplored.

Macrophage phenotype and metabolism are tightly interconnected^25^. M1-like macrophages show increased glycolysis, inducible nitric oxide synthase, and secrete pro-inflammatory cytokines. On the other hand, M2-like macrophages have increased mitochondrial respiration, higher arginase-1 expression, and the production of anti-inflammatory cytokines^26^. While recent reports have highlighted the potential role of macrophages in inflammaging by maintaining a pro-inflammatory phenotype^27^, the relationship between age-driven changes in metabolism, epigenetic signatures, and macrophage functions remains to be established. Prior research on age-dependent alterations in human and mouse macrophages has indicated increased monocyte levels, a potential reduction in phagocytic activity, and increase in inflammatory mediators with aging^28–31^. A recent study in human monocyte-derived macrophages identified MYC and USF1 as key regulators of macrophage dysfunction with aging^32^. However, a comprehensive, mechanistic framework for how aging reshapes macrophage biology remains lacking.

Here, we define macrophage aging as an active process of cellular reprogramming rather than the prevailing view of a gradual loss of function. Using mouse bone marrow-derived macrophages, we integrate transcriptomic, proteomic, and functional analyses to map the impact of aging across molecular and cellular layers. This approach reveals that aging reorganizes macrophage identity through coordinated changes in gene regulation, signaling networks, and effector functions, establishing a new conceptual model for how innate immunity is rewired over the lifespan.

## RESULTS

### Macrophages exhibit aging hallmarks and senescence phenotype.

**Figure 1:**
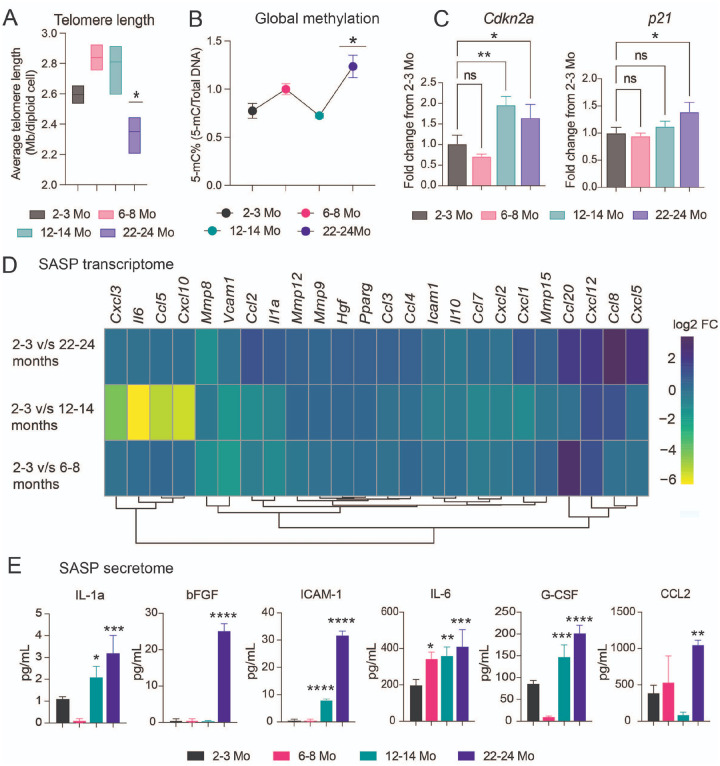
Aging hallmarks in macrophages. DNA isolated from bone marrow-derived macrophages of 2–3, 6–8,12–14, and 22–24 months old C57BL/6 mice was applied in assays to quantify **(A)** absolute telomere length, **(B)** changes in global DNA methylation, and **(C)** changes in global histone modification. **(D)**Changes in expression of genes associated with senescence-associated secretory phenotype (SASP) were determined 6–8,12–14, and 22–24 months in bone marrow-derived macrophages in comparison to 2–3 months-old macrophages (n=3). **(E)** The levels of select SASP proteins secreted in cell culture supernatant of bone marrow-derived macrophages of 2–3, 6–8, 12–14, and 22–24 months old as determined by microarrays (n=4). The *P* values were derived from a one-way ANOVA test. *P< 0.05, **P<0.01, ***P<0.001, ****P< 0.0001.

Our understanding of immuno-senescence is largely based on observed changes in lymphoid populations, such as T- and B-cells, with limited knowledge of myeloid cells, including macrophages. To establish aging hallmarks in macrophages, we investigated changes in telomere length, global methylation patterns, and senescence markers. DNA from bone marrow-derived macrophages (BMDMs) of four age groups (2–3, 6–8, 12–14, and 22–24 months) was used in quantitative PCR and colorimetric assays to determine changes in telomere length and global methylation, respectively. These age groups were chosen based on previous literature comparing mouse to human age^33^. Compared with the youngest group, 22–24-month-old macrophages had significantly shorter telomere length (Fig. 1A); however, telomere length did not differ significantly in the other age groups. Similar observations of shorter telomere length in old compared to young were reported for other cell types^34–36^. Aged macrophages (22–24-month-old) also showed a significant increase in methylation compared to 2–3-month-old macrophages (Fig. 1B). While the global 5-mC level can decrease with aging, hypermethylation is present in specific CpG islands^37,38^. These changes, however, can differ among different cell types, and previous studies have reported hypermethylation of genes associated with autophagy in aging macrophages^39^.

Using BMDMs of the same four age groups, we examined changes in transcript levels of well-known aging markers, such as *Cdkn2a* (*p16INK4a*) and *p21*. As expected, the expression of both aging-associated markers significantly increased in older macrophages (Fig. 1C), confirming the aging signature. Senescence-associated secretory phenotype (SASP) is considered a characteristic feature of cellular aging in macrophages and can potentially drive several age-related conditions^40^. Several studies have described a list of cytokines, chemokines, and growth factors considered part of the SASP^17,41^; however, there is no uniform consensus on a set of proteins driving SASP. Based on the literature describing common SASP proteins^17,19^, we used bulk RNA sequencing data to determine the changes in SASP genes with aging relative to the youngest group (Fig. 1D). Select cytokines, chemokines, growth factors, and proteases, such as *Ccl8*, *Cxcl5*, *Ccl2*, *Mmp12*, *Ccl3*, *Il1a*, *Cxcl1*, *Vcam1*, and *Hgf*, showed increased expression in 22–24-month-old macrophages. Other SASP genes either showed changes only in 12–14-month-old macrophages or did not change with age. To confirm these observations at the protein level, we used data from a multiplex ELISA assay that included select SASP proteins. Interestingly, the secreted levels of select SASP proteins (Il1α, Ccl2, Il6, Icam1, bfgf, G-csf) increased with aging (Fig. 1E), partially matching the transcriptome data. Other SASP proteins either decreased or remained unchanged with aging (**Supplementary Fig. 1A**). To compare SASP gene expression in human monocyte-derived macrophages, we used a publicly available RNA sequencing dataset (GSE100905) from donors 35 years or younger and 55 years or older. *CXCL10*, *CCL2*, *CXCL12*, and *CCL8* showed a significant increase in expression in older macrophages compared to younger macrophages (**Supplementary Fig. 1B**). While the age groups and the number of donors available in this dataset are limited, these findings validate the observation from mouse transcriptomic analysis of SASP. These results confirmed aging hallmarks in BMDMs.

### Aging disrupts macrophage phenotypic fate and signaling profiles.

**Figure 2:**
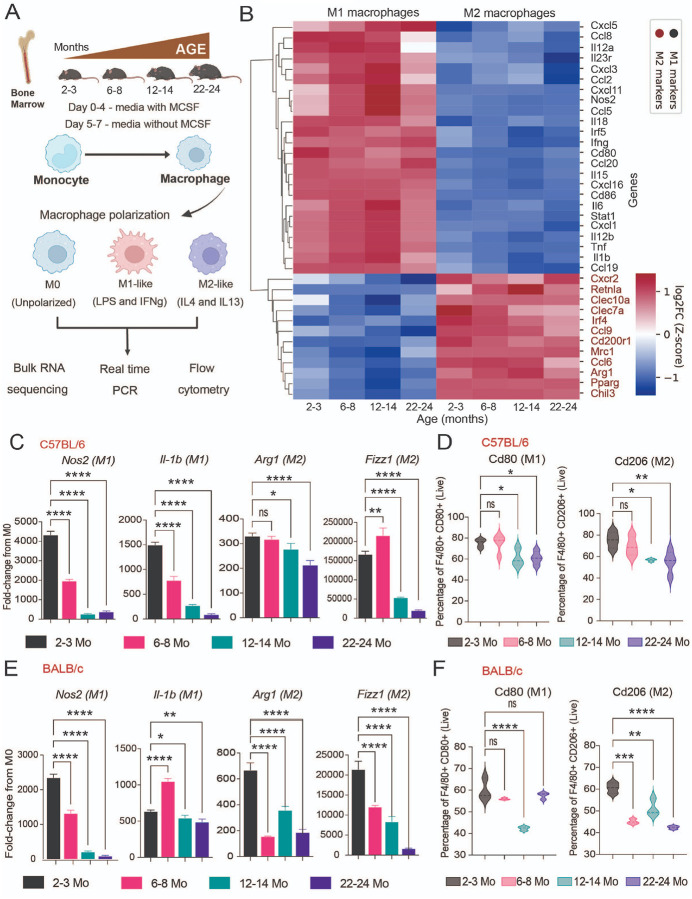
The transcriptome profile of genes associated with macrophage polarization is reduced with aging. **(A)** Bone marrow-derived macrophages from 2–3, 6–8, 10–12, and 22–24 months old C57BL/6 and BALB/c mice were polarized to M1- and M2-like phenotypes. **(B)** Bulk transcriptome analysis of bone marrow-derived macrophages highlighting the changes in expression of select M1 and M2 macrophage markers with age (n=3, biological replicates). **(C, E)** qPCR analysis of changes in gene expression of M1 and M2 macrophage markers are represented for C57BL/6 and BALB/c mice (n=3, technical replicates). **(D, F)** Flow cytometry analysis of CD80 and CD206, markers for M1 and M2 macrophages, respectively, demonstrated a defect in macrophage polarization with aging (n=3, technical replicates). The *P* values were derived from one-way ANOVA. *P< 0.05, **P<0.01, ***P<0.001, ****P< 0.0001.

To further characterize age-associated changes in macrophage phenotype, we used bulk RNA sequencing data of BMDMs isolated from C57BL/6 mice of four age groups (2–3, 6–8, 12–14, and 22–24 months) polarized to M1-like (with LPS and Ifnγ) or M2-like (with Il4 and Il13) phenotypes (Fig. 2A). Unpolarized BMDMs (M0) from each age group served as control samples. A clustermap showing the Z-score-normalized log2foldchange in transcriptome levels of select M1- (highlighted in black) and M2-like (highlighted in red) macrophage markers were used to compare changes across age groups (Fig. 2B). This list of markers was curated based on our previous macrophage transcriptomic data and literature review^10^. A clear and distinct separation in gene expression between M1- and M2-like macrophages confirmed successful polarization. In M1 macrophages, compared with 2–3 months, select genes (for example, *Ccl2*, *Cxcl11*, *Nos2*, and *Ccl5*) upregulated at 12–14-months and declined thereafter, indicating a nonlinear change with aging. While the expression of *Ccl8*, *Il12a*, *Il23r*, *Ccl2*, *Il18*, *Cd80*, and *Irf5* significantly reduced in 22–24-month-old M1 macrophages, *Cxcl5*, *Ifng*, *and Ccl20* showed upregulated gene expression with aging. In M2-like macrophages, the most downregulated expression with aging was observed for *Ccl9*, *Ccl6*, *Clec7a*, *Irf4*, and *Cd200r1* between 2–3- and 22–24-month-old macrophages.

To validate if similar age-associated changes are observed in routinely used M1- and M2-like macrophage markers, we looked at the relative gene expression changes of *Nos2, Il1b* (M1 markers), and *Arg1, Fizz1* (M2 markers) in a qPCR assay. Compared with the unpolarized controls of the respective age groups, the fold-change in expression of these M1- and M2-like markers significantly reduced with aging (Fig. 2C). Interestingly, the reduced expression changes with aging for these genes were more dramatic in qPCR when compared to bulk transcriptome data. The age-related changes were further reflected in the decline in the percentage of M1- and M2-polarized macrophages with aging, as assessed by flow cytometry analysis of cell-surface markers Cd80 and Cd206, respectively (Fig. 2D). To further confirm that these observations are not restricted to a specific mouse strain, we analyzed M1- and M2-like markers using BMDMs from BALB/c mice. We observed similar changes in qPCR (Fig. 2E) and flow cytometry (Fig. 2F) analyses, confirming that the response to cytokines associated with M1- and M2-polarization is significantly weaker at the transcript level in older macrophages. As a preliminary validation in human macrophages, we isolated monocyte-derived macrophages from donor primary blood mononuclear cells of four age groups (n = 2), 18–30, 30–50, 50–70, and above 70 years old, to determine changes in expression of M1- and M2-like macrophage markers. Similar to mouse BMDMs, the response of human monocyte-derived macrophages to M1- and M2-like polarization cytokines reduced with age (**Supplementary Fig. 1C**). To determine whether reduced response to polarization in aged macrophages is due to decline in cell-surface receptors associated with their polarization, we examined these receptors using flow cytometry. The percentages of M1 (Ifngr and Tlr4) and M2 (Il4r and Il13r) polarization receptors did not show baseline declines in unpolarized macrophages with age (**Supplementary Fig. 1D**). We also examined changes in the expression of recently described transcription factors, Myc and Usf1, in macrophages across ages. Validating the observations of Moss et al., *Myc* expression decreased in macrophages at 22–24 months, whereas *Usf1* did not show a significant change (**Supplementary Fig. 1E**). Overall, we observed that at transcript level, aged macrophages have reduced response to M1 and M2 polarization cytokine cues when compared to younger macrophages.

**Figure 3:**
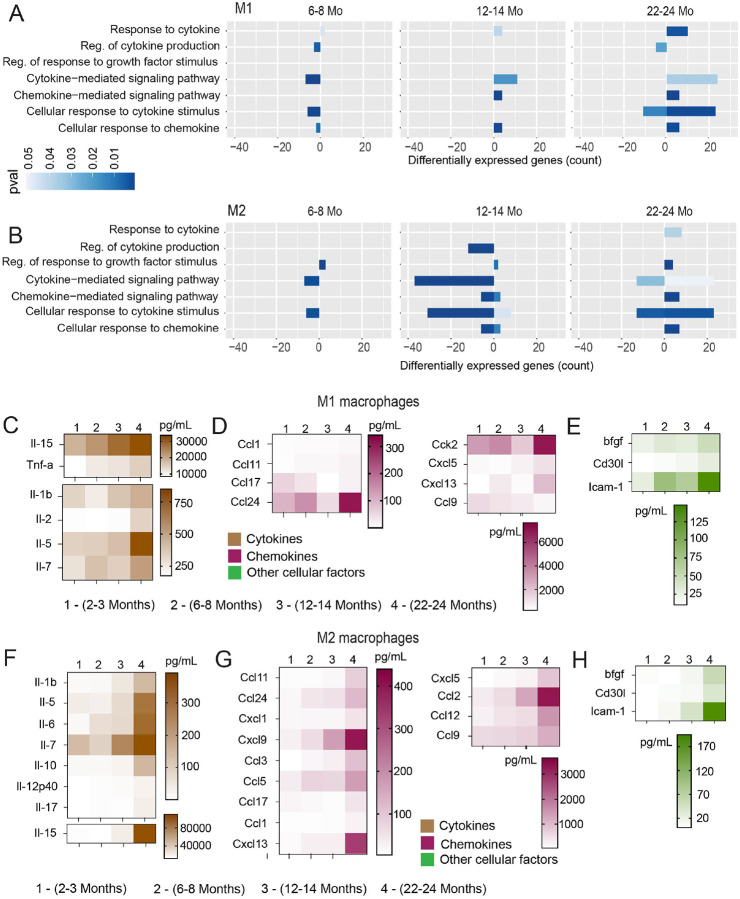
Age-associated changes in macrophage signaling pathways and secretome. Bulk RNA sequencing data from bone marrow-derived macrophages (n=3, biological replicates) across four age groups, polarized to M1 and M2 phenotypes, showed changes in pathways associated with cytokines, chemokines, and growth factors, as determined by KEGG pathway analysis. The x-axis count shows the number of genes within that specific pathway that are either upregulated or downregulated when compared to 2–3-month-old **(A)** M1 or **(B)** M2 macrophages. The cell culture supernatants from M1- and M2-polarized bone marrow-derived macrophages were applied to a mouse cytokine array to determine changes in the macrophage secretome profile with aging. The targets showing significant changes with aging are represented as **(C, F)** cytokines, **(D, G)**chemokines, and **(E, H)** growth and other factors.

Building on these observations, we further examined the effect of aging on signaling pathways relevant to macrophage phenotype. The Gene Ontology (GO) enrichment analyses on bulk RNA sequencing data revealed that in M1-like macrophages, aging significantly changes the expression of genes associated with pathways of cytokine production, response, and signaling, especially in 22–24-month-old macrophages, along with modest changes to chemokine pathways (Fig. 3A). Interestingly, in M2-like macrophages, we observe significant downregulation of genes related to cytokine and chemokine pathways in 12–14-month-old macrophages. However, in 22–24-month-old macrophages, the genes associated with these signaling pathways are either significantly upregulated or downregulated (Fig. 3B). The macrophage secretome is essential for both homeostasis and driving appropriate immune responses. Taking cues from bulk RNA transcriptomic data, we quantified changes in cytokine (brown), chemokine (magenta), and other cellular factor (green) secretion using a Mouse Cytokine Array (RayBiotech). M1-polarized macrophages secreted higher levels of pro-inflammatory cytokines (Fig. 3C), chemokines (Fig. 3D) and growth factors (Fig. 3E) with aging. Levels of Ccl17 and Ccl9, key mediators of fibrosis and anti-inflammatory phenotype, respectively, reduced with age (Fig. 3D). A key observation is that aged M2-like macrophages also secrete increased amounts of pro-inflammatory cytokines (Fig. 3F), chemokines (Fig. 3G), and other cellular factors (Fig. 3H), despite polarized to anti-inflammatory phenotype. These secretome profiles of aged macrophages show an inflammatory signature, supporting the role of macrophages in “inflammaging”. Overall, these results demonstrate that, in addition to a poor response to polarizing cytokines, aged macrophages maintain an inflammatory phenotype.

### The epigenetic signature of macrophages is remodeled with aging.

**Figure 4:**
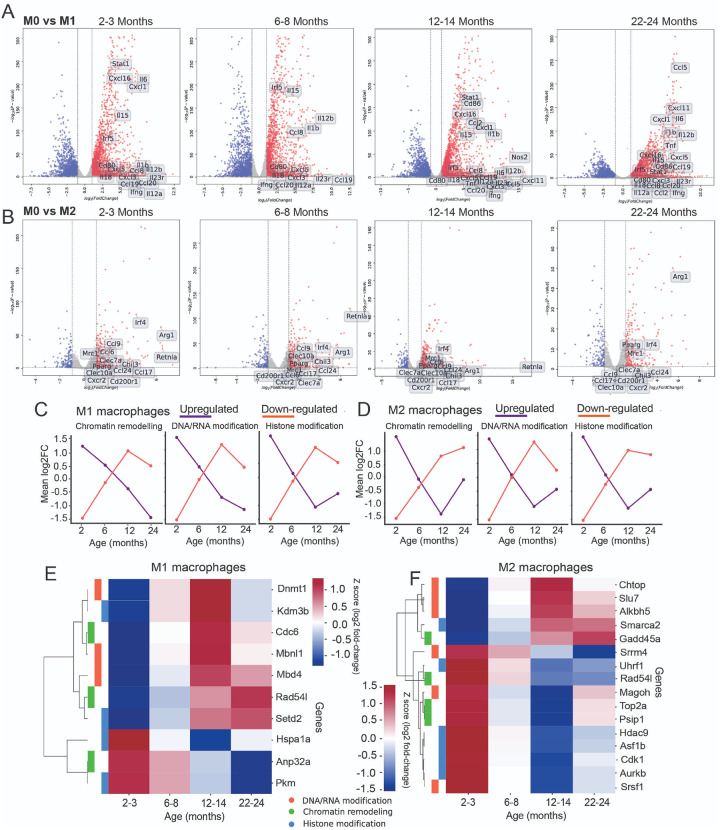
The epigenetic landscape of macrophages changes with aging. The volcano plots showing differentially expressed genes in **(A)**M1- or **(B)** M2-polarized bone marrow-derived macrophages from four age groups, compared with unpolarized M0 samples of the respective ages. The kinetics of changes (increase or decrease) in expression of genes associated with epigenetic modifications, including DNA/RNA modification, chromatin remodeling, and histone modification, with age in **(C)** M1- and **(D)** M2-like macrophages. Cluster map representing changes in expression of select epigenetic modifiers with age in **(E)** M1- and **(F)** M2-like macrophages as an example. The statistic parameters and test from PyDESeq2 calculates the *P*value using a Wald test within a negative binomial generalized linear model adjusted using the Benjamini–Hochberg false discovery rate (FDR) procedure, the signfiicance is adjusted *P* value; P < 0.05 for nominal significance, P < 0.01 for stronger evidence, with FDR < 0.05.

The role of epigenetic regulation in macrophage differentiation, activation, and phenotypic plasticity has been extensively documented^42,43^, but the age-related epigenetic changes in macrophages have not been previously addressed. Given the observed changes in global DNA methylation, we next investigated whether aging also reshapes specific epigenetic changes in macrophages. Bulk transcriptomic data were used to identify changes in genes associated with epigenetic regulation. Volcano plots comparing polarized macrophages to unpolarized M0 controls revealed an age-associated global reduction in gene expression in both M1 (Fig. 4A) and M2 (Fig. 4B) conditions. When focusing specifically on genes involved in chromatin remodeling, DNA/RNA modifications, and histone modifications, we observed both upregulation and downregulation with aging (Fig. 4C–D), indicating that age-related epigenetic remodeling is not unidirectional but rather dynamically regulated.

Among significantly altered epigenetic regulators in aged macrophages, the majority were associated with histone modification pathways (highlighted in blue in Fig. 4E–F and **Supplementary Fig. 2A–B**). For example, in M1 macrophages, genes such as *Hspa1a, and Pkm* were downregulated while *Kdm3b and Setd2* expression increased with aging (Fig. 4E). Similarly, in M2 macrophages, with aging, significant decline in expression of *Uhrf1, Hdac9, Asf1b, Cdk1, and Aurkb* was observed along with increased expression of *Smarca2* (Fig. 4F). Notably, there was minimal overlap between age-regulated epigenetic targets in M1 and M2 macrophages, suggesting that aging differentially reshapes the epigenetic signature of distinct macrophage phenotypes, potentially leading to divergent functional consequences.

**Figure 5:**
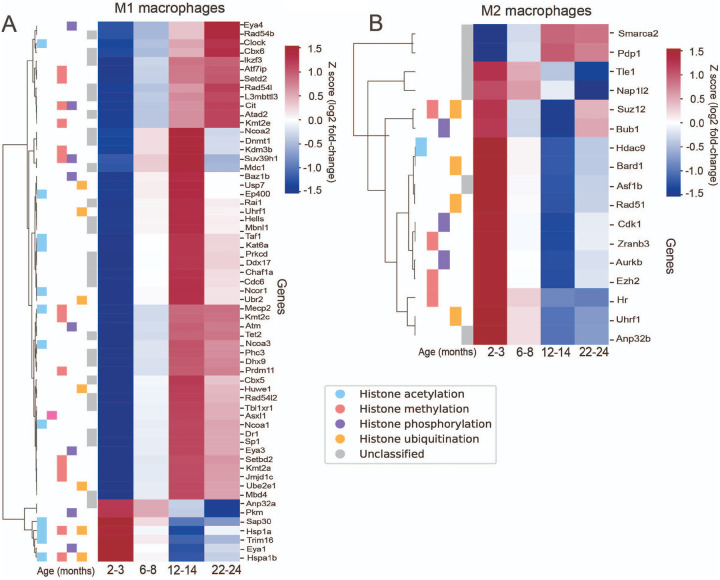
Aging macrophages show significant changes in histone modifications. Cluster maps representing the changes in different histone modifications with aging in **(A)**M1 and **(B)** M2 polarized bone marrow-derived macrophages.

The focus on genes specifically involved in histone modifications, including methylation, acetylation, phosphorylation, and ubiquitination, revealed significant age-associated alterations in both M1- and M2-like macrophages (Fig. 5). In M1-like macrophages, we noticed that genes such as *Setd2*, *Kmt2e*, *Jmjd1c*, *Eya4*, and *Cbx6* to name a few that are known to affect macrophage polarization and function were upregulated, whereas *Sap30*, *Trim16*, and *Hspa1b* involved in regulating inflammation and metabolic activity of macrophages were downregulated with age (Fig. 5A). In contrast, M2-like macrophages displayed a predominant trend toward downregulation of histone modification-associated genes with aging (Fig. 5B). This largely included genes associated with anti-inflammation (*Uhrf1, Ezh2*), fibrosis (*Tle1, Nap1l2*), and DNA damage response (*Bub1*, *Bard1*, *Rad51*, *Cdk1*, *Aurkb*, *Zranb3)*. To clarify that several genes participate in multiple histone modification processes, we color-coded each category (acetylation, methylation, phosphorylation, and ubiquitination) in the cluster maps. Genes represented in gray are not yet assigned to a specific histone modification category in the current database. These changes, especially with genes involved in histone modifications, indicate that aging significantly alters the epigenetic signature that can directly alter macrophage phenotype and functions. Future studies aim to validate the impact of these age-associated changes in histone modification on their target proteins.

### Aged macrophages have an altered metabolic gene signature, with increased lipid activation and transport pathways and decreased mitochondrial redox- and cholesterol-associated pathways.

**Figure 6:**
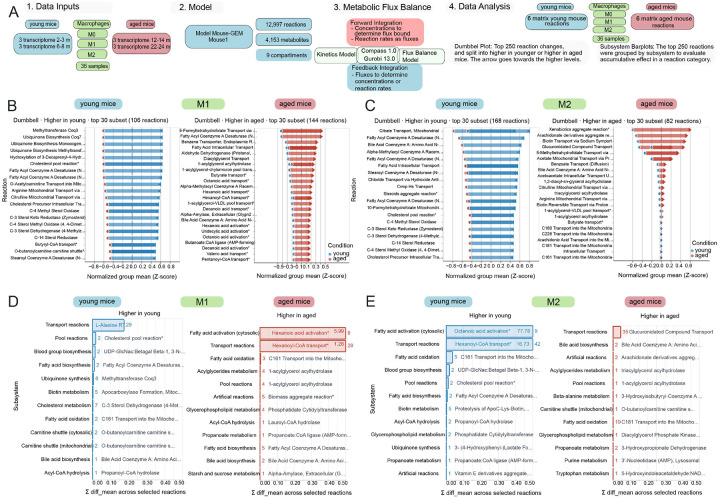
Age-driven remodeling of macrophage metabolic flux landscapes inferred from transcriptome-constrained genome-scale modeling. **(A)** Overview of the computational workflow used to infer metabolic flux differences between macrophages from young (2–3 and 6–8 months) and aged (12–14 and 22–24 months) mice. Transcriptomic profiles were stratified by macrophage polarization state. Gene-expression data were integrated with the Mouse-GEM genome-scale metabolic reconstruction. Flux balance estimation was performed using Compass 1.0 with Gurobi 13.0, followed by forward and feedback integration steps to identify reactions with increased or reduced inferred activity between age groups. **(B)** Reaction-level dumbbell plots showing selected subsets of the top age-associated flux differences in M1 and M2 macrophages. Each point represents the normalized group mean flux score for young or aged mice, expressed as a Z-score, with connecting arrows indicating the direction of increased inferred reaction activity. Reactions were separated into those higher in young mice and those higher in aged mice. **(C)** Subsystem-level aggregation of the top 250 differential reactions between young and aged macrophages. Bars represent the cumulative summed mean difference across selected reactions within each metabolic subsystem, with adjacent labels indicating representative reactions contributing to each category. Together, these analyses reveal polarization-specific and age-dependent redistribution of macrophage metabolic flux potential, with aging associated with a shift toward lipid activation, transport, and remodeling pathways. The statistics values from the top 250 differential reactions were for Cohen’s d effect sizes ranged from −1.54 to 1.61 (Z-score), with a median of −0.18. In the inferential analysis, *P*values ranged from 0.0209 to 1.0, with 25 significative reactions (*P*<0.05).

We next examined whether the age-associated changes in macrophages observed so far in the above experiments are potentially accompanied by a coordinated shift in metabolic signatures. We applied transcriptome-constrained genome-scale metabolic modeling using Compass^44^ and the Mouse-GEM^45^ metabolic reconstruction to bulk RNA-seq profiles from BMDMs. For this analysis, transcriptome data from 2–3- and 6–8-month-old macrophages were grouped as young, whereas 12–14- and 22–24-month-old macrophages as aged, to increase the sample size and enable reaction-level comparison of inferred metabolic activity. The workflow is summarized in Fig. 6A. The flux landscapes showed that macrophage aging was not associated with a generalized collapse of metabolic activity, but rather with a redistribution of inferred metabolic flux potential. In the main flux balance analysis, aged M1 macrophages showed increased inferred activity in pathways related to fatty acid activation and acyl-CoA, whereas in aged M2 macrophages, glucuronidated compound transport, arachidonate-related reactions, acylglyceride metabolism, and tryptophan-associated pathways were more prominent (Fig. 6B–C). In contrast, young macrophages retained higher inferred activity in mitochondrial transport, cholesterol-associated reactions, ubiquinone biosynthesis, fatty acid oxidation, and selected pool or transport reactions (Fig. 6B–C).

The subsystem-level aggregation further supported this interpretation. Among the top differential reactions (Fig. 6D–E), aged macrophages showed dominant cumulative signals in fatty acid activation, transport reactions, acylglyceride metabolism, glycerophospholipid metabolism, and xenobiotic/glucuronidation-associated transport. By contrast, young macrophages were enriched for transport reactions, pool reactions, cholesterol metabolism, fatty acid biosynthesis, fatty acid oxidation, ubiquinone synthesis, and mitochondrial carnitine shuttle activity. Thus, aging shifts the macrophage metabolic signature towards lipid activation, lipid trafficking, and membrane remodeling programs, while reducing a coordinated mitochondrial-redox and lipid-oxidative configuration.

To determine whether these flux signatures were restricted to M1 and M2 macrophages or reflected a broader age-associated metabolic program, we combined the macrophage dataset from M0, M1, and M2 polarization states. This supplementary analysis confirmed that age-associated flux remodeling was detectable across macrophage states, although the specific reaction categories differed by polarization (**Supplementary Fig. 3**). Finally, to connect the metabolic flux-level results with the broader aging phenotypes observed so far in this study, reaction-associated genes were mapped to curated categories related to SASP, macrophage polarization, histone modification, and DNA methylation. This analysis showed that age-associated metabolic reactions overlapped with gene programs linked to inflammatory remodeling, altered polarization, and epigenetic regulation (**Supplementary Fig. 4**). Overall, these transcriptomic analyses indicated an age-dependent alteration in macrophage metabolism, which will be validated in the future through mass spectrometry experiments.

### Aged macrophages exhibit enhanced phagocytic capacity, diminished migratory ability, and preserved antigen presentation.

**Figure 7:**
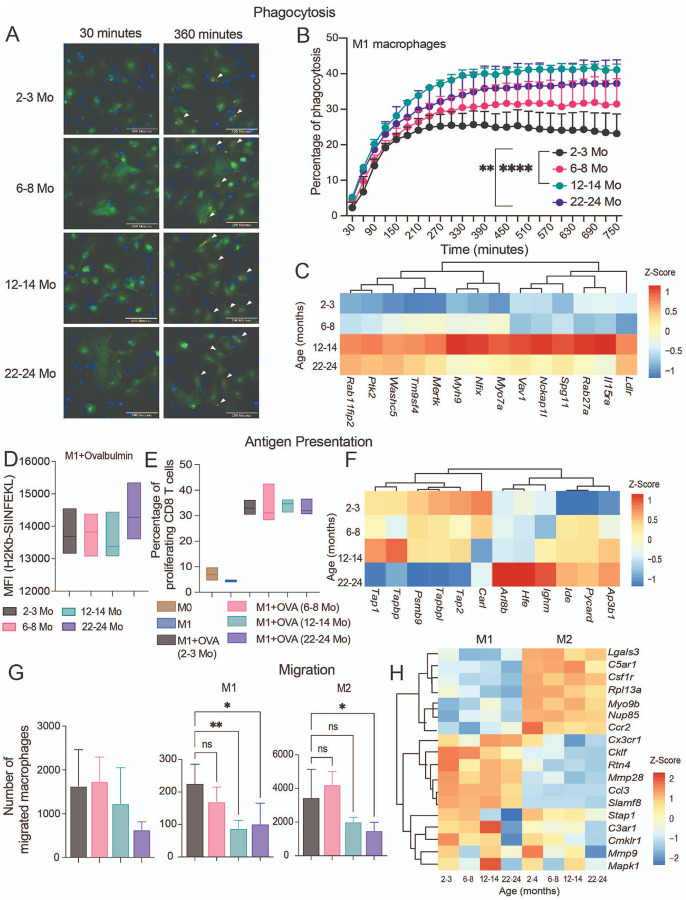
Macrophage functions are affected by aging. Bone marrow-derived macrophages from UBC-GFP mice aged 2–3, 6–8, 12–14, and 22–24 months were polarized to M1 macrophages and co-cultured with SM1 melanoma tumor cells. **(A)** Change in percentage of phagocytosis with age, **(B)** the representative images, and **(C)**phagocytosis-associated gene signature. Wild-type C57BL/6 bone marrow-derived macrophages from different age groups polarized to M1 macrophages were incubated with ovalbumin. **(D)** The mean fluorescence intensity of cells expressing the H2-kb-SIINFEKL. Ovalbumin-pulsed M1 macrophages from four age groups were co-cultured with T cells isolated from OT1 mice and stained with Cell Trace Violet. **(E)** The percentage of proliferating CD8 T-cells. **(F)**The changes in expression of genes related to antigen presentation. UBF-GFP mice from the same four age groups were seeded in the top well of the transwell system, polarized to an M1 or M2 phenotype, and co-cultured with SM1 melanoma tumor cells in the bottom well. **(G)** The number of M0, M1, and M2 macrophages migrating to the bottom well was quantified. **(H)** The changes in expression of macrophage migration-related genes that decrease with aging. The *P* values were derived from one-way ANOVA. *P< 0.05, **P<0.01, ***P<0.001, ****P< 0.0001.

Macrophages are well-established phagocytic cells that recognize, engulf, and remove foreign pathogens, cellular debris, and cancer cells^46^. Previous studies have reported an age-associated decline in macrophage phagocytic activity^28,32^. However, the majority of these studies have used fluorescent beads as targets rather than cells. Here, we co-cultured BMDMs from UBC-GFP mice across four age groups with tumor cells to measure phagocytic activity. Tumor cells stained with pHRodo dye when engulfed by macrophages emit fluorescence due to the low pH of phagolysosomes (Fig. 7A). We have previously established that M1-like macrophages have higher phagocytic activity when compared to M2-like polarized macrophages^12^. Upon co-culture with tumor cells, the percentage of phagocytosis was significantly higher in 12–14-, and 22–24-month M1-like macrophages compared to 2–3-month-old at different time points (Fig. 7A–B). The 12–14-month-old macrophages showed a significant increase in phagocytic activity starting at the 3-hours, whereas the 22–24-month-old around 5.5-hours after co-culture. The percentage of phagocytosis by the 22–24-month-old macrophages remained lower than that of the 12–14-month-old, indicating that the phagocytic activity potentially declines beyond a certain age. We applied transcriptomic data from M1-polarized macrophages to examine gene expression changes associated with phagocytic activity (GO:0006909) (**Supplementary Fig. 5A**). Based on this curated list, we identified genes that matched the observed age-associated changes in phagocytosis (Fig. 7C). This gene signature included *Ldlr, Il15ra*, *Rab27a*, *Spg11*, *Nckap1l*, *Vav1*, *Myo7a*, *Nfix*, *Myh9*, *Mertk*, *Tm9sf4*, *Washc5*, *Ptk2*, and *Rab11fip2*, which are mainly involved in phagosome formation and phagocytic function.

Dendritic cells and macrophages are the professional antigen-presenting cells, along with B cells. This function is essential for mounting appropriate adaptive immune responses. The impact of aging on the antigen-presenting ability of macrophages is unclear, as the current literature yields conflicting results^31,47,48^. To determine changes in the antigen presentation machinery with aging, M1-like macrophages from different age groups were incubated with ovalbumin and analyzed for the presentation of the SIINFEKL peptide on the H-2Kb MHC class I complex. While there was a trend towards higher antigen presentation by 22–24-month-old macrophages, this difference was not significant compared with the youngest age groups (Fig. 7D). To further determine whether there are changes in antigen cross-presentation, we co-cultured ovalbumin-treated M1-like macrophages from four age groups with Cell Trace Violet (CTV) dye-stained T cells isolated from 2–3-month-old OT-1 mice (that express a transgenic CD8 + T-cell receptor specific for ovalbumin). CD8 + T-cell proliferation was determined based on the diluted intensity of CTV dye using flow cytometry. While the percentage of proliferating CD8 + T-cells was significantly higher when co-cultured with M1-like macrophages pulsed with ovalbumin, macrophage age itself did not significantly affect proliferation (Fig. 7E). Overall, the macrophage antigen presentation machinery appears to be preserved with aging. The transcriptome data, however, showed differential expression of genes involved in antigen processing and presentation (GO:19882) with age (**Supplementary Fig. 5B**), including a reduction in genes like *Tapbp*, *Tap1*, *Tap2*, *Tapbpl*, *Psmb9*, *Calr*, that are part of the MHC-I chaperone and antigen cross-presentation, and an increase in *Arl8b, Hfe, Ighm, Ide, Ap3b1, and Pycard* that participate in intracellular trafficking, as adaptor molecules and regulators of antigen presentation (Fig. 7F).

Macrophage migration plays a crucial role in tissue repair and tumorigenesis. Macrophages are known to migrate in response to various stimuli, mainly driven by differential gradients. To determine whether age affects macrophage migration, we co-cultured GFP+ M0, M1, or M2 polarized macrophages (in the top insert) with tumor cells (in the bottom well) in a transwell system and quantified the number of GFP+ macrophages that migrated to the bottom well. Migration of both M1- and M2-like macrophages significantly decreased with aging, especially in 22–24 months (Fig. 7G). The number of M2-like macrophages migrating to the bottom well was higher in all age groups when compared to the M1-like macrophages. While the change in migration of control, M0, macrophages with age was not significant, it showed a trend of reduction with age (Fig. 7G). At the transcript level, we analyzed changes in expression of genes specific to macrophage migration using the MGI gene ontology database (GO:1905517) (**Supplementary Fig. 5C**). When we narrowed down the list to genes that were downregulated with aging in M1- and M2-like macrophages (Fig. 7H), the signature included genes that are critical for chemotaxis (*Cx3cr1*, *Lgals3*, *C5ar1*, *Mapk1, Ccl3, Cklf*), tissue infiltration (*C5ar1, Nup85, Rtn4, C3ar1*), macrophage motility (*Rpl13a, Myo9b*), recruitment (*Mmp28, Cmklr1*), receptors driving migration (*Csfr1, Ccr2*), and degradation of ECM (*Mmp9*).

## DISCUSSION

The current understanding of the aging immune system, defined by reduced effector functions and a decline in clonal diversity, is largely shaped by observations in adaptive immunity, including T- and B-cells, without capturing in detail the impact on innate immune cells^35,49^. Despite their prominent role in homeostasis, tissue repair, and direct involvement in numerous age-related chronic diseases^2,28,50^, the impact of aging on macrophages is understudied. Previous reports show changes in the proportion of monocytes/macrophages with aging (myeloid skewing and monocytopoiesis in bone-marrow, for example), the role of tissue-resident macrophages in age-specific conditions, and the contribution of macrophages towards “inflammaging”^32,40,50,51^. Recent studies propose transcription factors involved in macrophage functional defects with aging and potential mechanisms behind macrophage senescence^32,40^. Beyond these encouraging observations, a more integrated approach is needed to define the molecular and functional changes associated with macrophage aging. In this study, we demonstrate, for the first time, that aging progressively drives macrophage dysfunction through coordinated changes in cytokine and chemokine signaling pathways, phenotypic polarization, and the metabolic and epigenetic landscape, with low-grade inflammation in the backdrop.

In bone marrow-derived macrophages (BMDMs) isolated from mice across four age groups (2–3, 6–8, 12–14, and 22–24 months), aging hallmarks were evident with a decrease in telomere length, an increase in global DNA methylation, and higher expression of senescence markers, Cdkn2a and p21, in aged macrophages. The elevated levels of proteins and gene expression related to senescence-associated secretory phenotype (SASP) in older macrophages further corroborated the contribution of macrophages to “inflammaging” and its potential role in age-associated diseases, including atherosclerosis, macular degeneration, lung fibrosis, and skeletal muscle injury^17,18,52^. Macrophage phenotype and plasticity are driven by cytokine cues in the surrounding environment. When exposed in vitro to classical M1- (LPS and Ifn-γ) and M2-like (Il4 and Il13) polarization cytokines, aged macrophages responded poorly based on the significant decrease in routinely used M1 and M2 macrophage markers at transcriptome and cell surface receptor levels. This further supports previous observations of a prolonged inflammatory phase and slower wound healing in the older population due to failed phenotype switching in aged macrophages^53^. Preliminary experiments in human monocyte-derived macrophages from donors across four age groups showed a similar reduction in polarization capacity with aging.

Gene Ontology analysis revealed significant age-driven changes in genes associated with cytokine and chemokine signaling pathways, and, irrespective of phenotype, aged macrophages secreted higher levels of inflammatory cytokines, chemokines, and other cellular factors. Notably, the elevated inflammatory secretome of aged macrophages polarized under anti-inflammatory (M2-like) conditions once again highlighted a shift towards low-grade inflammation with aging and a potential deficit in inducing the anti-inflammatory pathways. At the molecular level, the epigenetic and metabolic signatures were dynamically altered with aging. The transcriptome profile of genes associated with key epigenetic processes varied with age and showed substantial changes to histone modifications. For instance, genes like *Setd2*, *Ktm2e*, and *Tet2*, to name a few, are upregulated in older M1 macrophages, suggesting a mechanism for regulating excessive inflammation with aging. In contrast, we observe that genes like *Uhrf1*, *Nap1l2*, and Ezh2, which promote anti-inflammation and fibrosis, are downregulated with age, indicating a defective M2-like phenotype at the epigenetic level. The changes in the signatures of histone-modifying genes with aging are unique between M1- and M2-polarized macrophages, emphasizing that a one-size-fits-all theory does not apply to all macrophage phenotypic states. Aging also reprograms macrophage metabolic state rather than inducing exhaustion, as observed in T- and B-cells. Based on the Compass–Mouse-GEM analysis, aged macrophages retain active metabolic programs, but these programs are redistributed toward lipid activation, acyl-CoA transport, acylglyceride remodeling, and xenobiotic/glucuronidation-linked transport, but away from mitochondrial-redox and cholesterol-associated programs. These results are consistent with the concept that inflammaging reflects an immune–metabolic state in which chronic inflammatory cues, altered nutrient handling, and cellular stress responses become coupled during aging^20,54^. The flux balance analysis further provided a mechanistic layer linking macrophage aging hallmarks with metabolic programs that may support altered cytokine secretion, lipid remodeling, phagocytic adaptation, and reduced migratory capacity.

Finally, the phenotypic and molecular changes with aging directly reflected on macrophage functions. Here, we demonstrate that macrophage phagocytic activity increases with age, whereas their migratory ability is significantly reduced. These findings correlate with the enhanced inflammatory phenotype described above, with higher phagocytic activity observed in macrophages from 12–14- and 22–24-month-old individuals, and reduced migration, particularly in M2-like macrophages. This divergence highlights a functional imbalance in aged macrophages, characterized by preserved or enhanced local activity (like phagocytosis) but impaired dynamic (or migratory) responsiveness. Using Gene Ontology databases, we identified, for the first time, a macrophage function-specific gene signature that closely matches the functional changes observed with aging. This provides an important framework for future studies aimed at determining whether targeted modulation of these gene programs can alter macrophage functions. Despite widespread transcriptional changes, the antigen-presenting machinery appeared largely conserved with aging, suggesting selective preservation of key immune functions.

**Figure 8:**
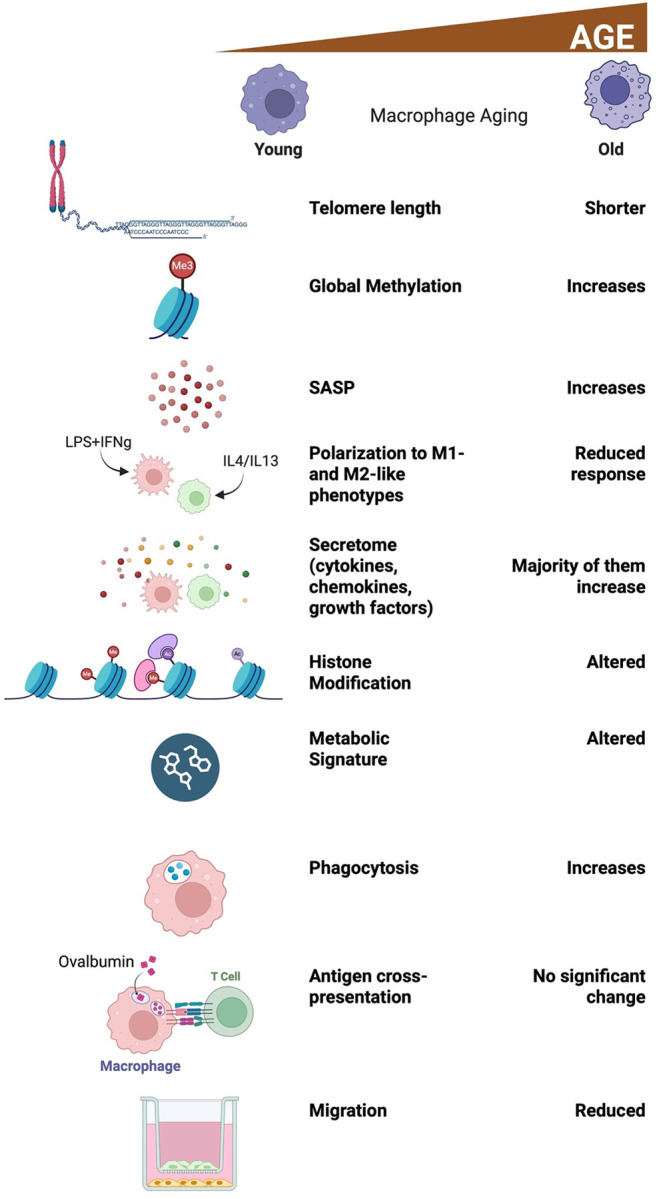
Schematic summary of the impact of aging on macrophage biology.

Overall, this study provides a comprehensive understanding of age-driven changes in macrophage biology at molecular, metabolic, and functional levels (Fig. 8). Given the central role of macrophages in homeostasis and in several age-associated chronic conditions, these findings establish a much-needed foundation for future investigations to understand and therapeutically target aged macrophages across diverse disease settings.

## MATERIALS AND METHODS

### Macrophage isolation, maintenance, and polarization

Bone marrow-derived macrophage (BMDM) isolation and polarization was performed as per previous optimized protocols^55^. Briefly, bone marrow was harvested from wild-type C57BL/6 mice of four age groups: 2–3, 6–8, 12–14, and 22–24 months. Cells were cultured in RPMI (Corning) (containing 10% FBS, 1% Pen-Strep, and 1% non-essential amino acids) with 20 ng/mL of macrophage colony-stimulating factor (M-csf, BioLegend) to differentiate the monocytes into macrophages. After four days, the cell culture media were replaced with M-csf-free complete RPMI and maintained for an additional three days before use in the experiment. Mouse BMDMs were polarized to M1 phenotype with 100 ng/mL of LPS and 50 ng/mL of Ifnγ (BioLegend) or to M2 phenotype with 20 ng/mL each of Il4 (BioLegend) and Il13 (BioLegend). Unpolarized M0 macrophages serve as baseline control.

Using Ficoll and density gradient centrifugation, primary blood mononuclear cells were isolated from leukocyte reduction system (LRS) chamber of donor blood obtained from INOVA Blood Donor Services (Sterling, Virginia). Monocytes were purified by following the manufacturer’s instruction of EasySep Human Monocyte Isolation Kit (StemCell Technologies). Monocytes were cultured in complete RPMI media (containing 10%FBS, 5% human serum, 1% Pen-Strep, and 1% non-essential amino acids) with 50 ng/mL of human M-CSF (BioLegend). Media was changed to M-CSF-free media after 4 days and maintained for additional 3 days before starting the experiments. Human monocyte-derived macrophages were polarized to M1-like phenotype with LPS (10 ng/mL) and IFNγ (50 ng/mL, BioLegend) and to M2-like phenotype with IL4 (10 ng/mL, BioLegend) and IL13 (10 ng/mL, BioLegend).

### Telomere and global methylation assay

BMDMs from wild-type C57BL/6 mice of four age groups were isolated and cultured as described above. After seven days of differentiation and maintenance, the cells were harvested for DNA isolation using the DNeasy kit (Qiagen) according to the manufacturer’s instructions. The DNA samples from macrophages of all four age groups were used in the Absolute Mouse Telomere Length Quantification qPCR Assay Kit (ScienCell) to quantify the telomere length. Briefly, 2 ng of DNA was used to measure the average telomere length of mouse macrophages using the telomere primer set. A single-copy reference primer set that recognizes and amplifies a 100-base pair long region on mouse chromosome 10 was used as a reference for normalizing the data. The isolated DNA was applied to the MethylFlash Global DNA Methylation ELISA kit (EpigenTek) to investigate changes in global DNA methylation associated with aging. Following the manufacturer’s protocol, 100 ng of DNA was added to the plate specifically treated for high DNA affinity. The capture and detection antibodies identify the methylated fraction of DNA and were quantified calorimetrically. The percentage of methylated DNA (5-mC) is proportional to the absorbance measured at 450 nm, which was further determined using the standard curve generated from the positive control provided in the kit.

### qPCR, flow cytometry, bulk RNA sequencing, multiplex ELISA

BMDMs from wild-type C57BL/6 and BALB/c mice of different age groups were polarized to M1 and M2 phenotypes as described above and harvested in TRIzol reagent (Invitrogen) 16 hours after polarization. The mRNA isolated by the phenol-chloroform method was converted to cDNA using the iScript cDNA Synthesis Kit (Bio-Rad). Changes in the expression of M1 (*Nos2, Il1β*) and M2 (*Arg1, Fizz1*) markers were quantified by real-time PCR with unpolarized M0 samples as control. cDNA from unpolarized samples across all age groups was used to quantify changes in the expression of age-related markers, such as *Cdkn2a* and *p21*. Human monocyte-derived macrophages were polarized for 24 hours before harvesting in TRIzol reagent for RNA isolation. BMDMs isolated from four age groups were polarized to M1 and M2 phenotypes for 24 hours, and cells were gently dissociated by scraping in non-enzymatic cell dissociation buffer (Gibco). Unpolarized cells (M0) served as a control. Following the Live/Dead Aqua Zombie staining (1:1000 dilution) in PBS for 20 minutes, the cells were washed in FACS buffer and stained with antibodies against F4/80 (pan macrophage marker), Cd80 (M1 macrophage marker), and Cd206 (M2 macrophage marker). Data acquired by flow cytometry were analyzed with FlowJo software, and changes in the percentages of F4/80 + Cd80+ and F4/80 + Cd206+ populations were determined. The gating strategy is shown in Supplementary Table 1. Information on primer sequences and flow cytometry antibodies is listed in Supplementary Table 2 and Supplementary Table 3, respectively.

For bulk RNA sequencing, BMDMs from four age groups of C57BL/6 mice (n = 3) were polarized into M1 and M2 macrophages, along with unpolarized M0 macrophages, for 16 hours and harvested in TRIzol reagent. Samples were submitted to Azenta Life Sciences for RNA sequencing. Raw transcript counts from triplicate samples were used as the input matrix for DESeq2. The standard DESeq pipeline^56^, using a Negative Binomial/Gamma Poisson distribution, was used for differential expression analysis. Comparisons between age and/or macrophage polarization were performed as appropriate to the experimental question. For expression of SASP-related genes, differentially expressed genes in unpolarized macrophages (M0) across age groups relative to the youngest (2–3 months) were used.

For general enrichment, differentially expressed genes between each age group and the youngest age group, within macrophage polarization states (e.g., 6–8 Mo M1 vs. 2–3 Mo M1), were divided into up- and down-regulated lists based on log2FC > 1.0 or < −1.0, respectively, and on an adjusted p-value < 0.05. These genes served as the input for the EnrichR package^57–59^, referencing the 2024 Gene Ontology Biological Processes (GO-BP) library^60,61^. The following terms associated with general cytokine, chemokine, and growth factor pathways were selected: “response to cytokine” (GO:0034097), “regulation of cytokine production” (GO:0001817), “regulation of cellular response to growth factor stimulus” (GO:0090287), “chemokine-mediated signaling pathway” (GO:0070098), “cytokine-mediated signaling pathway” (GO:0019221), “cellular response to cytokine stimulus (GO:0071345), cellular response to chemokine” (GO:1990869).

Cell culture supernatants from M0, M1, and M2 macrophages were collected after 24 hours of polarization. After centrifugation at 2000 *g* for 10 mins at 4°C, the conditioned media were aliquoted and stored at −80°C. The Quantibody Mouse Cytokine Array 5 (RayBiotech) was used to measure cytokine levels secreted by macrophages across different age groups and phenotypes. Following the manufacturer’s protocol, the signal from the slides was quantified. The Quantibody Mouse Cytokine Array (Ray Biotech) included 40 targets, several of which were relevant to the macrophage secretion profile. Cell culture supernatants from M1- and M2-polarized BMDMs and M0 controls were analyzed in a multiplex ELISA according to the manufacturer’s instructions. The average signal intensity from four technical replicates of each target was used to quantify the amount (pg/mL) of secreted protein, using a standard curve, followed by normalization to β-actin expression from the harvested cells.

### Epigenetic transcriptome analysis

This study employed a comprehensive computational workflow to process and perform downstream analysis of RNA sequencing (RNA-seq) data to evaluate gene expression dynamics across macrophage phenotypes with aging. All analyses were performed using established open-source bioinformatics pipelines, with statistical modeling and visualization conducted in R and Python to ensure transparency and reproducibility.

Paired-end RNA sequencing was performed on an Illumina platform, generating 150 bp reads (R1 and R2). Raw reads were processed using the nf-corernaseq pipeline (v3.21.0)^62^ implemented in Nextflow^63^ under a Docker environment to ensure computational reproducibility. The workflow included adapter trimming, alignment, and quantification. Reads were aligned to the Mus musculus GRCm39 reference genome (Ensembl release 112)^64^, and gene-level quantification was performed with Salmon in alignment-based mode. Transcript-level estimates were summarized to gene-level counts using Ensembl annotations, producing a merged count matrix (salmon.merged.gene_counts.tsv). Quality control (QC) of both raw and processed sequencing data was conducted to evaluate read quality, duplication rates, mapping distribution, and sample consistency before downstream analysis. Analyses focused on macrophages across four age groups (2–3, 6–8, 12–14, and 22–24 months). Genes exhibiting very low expression were filtered by retaining only those with a minimum of 10 total read counts across all samples.

Differential gene expression (DGE) analysis was performed using DESeq2^65^. Raw count data were modeled with a negative binomial generalized linear model to assess the effects of macrophage group, time, and their interactions. Gene Set Enrichment Analysis (GSEA) was applied to pre-ranked gene lists based on DESeq2 stat values to identify pathways associated with significant temporal trends. Analyses were performed against three gene set collections: KEGG, Gene Ontology (GO) Biological Process, and a custom epigenetic gene set. A detailed method is described in the supplementary materials, including all scripts, configurations, and processed data supporting this study.

### Genome-scale metabolic modeling and age-associated flux analysis

Transcriptome-derived metabolic states were reconstructed across macrophage polarization states and age groups using a Compass-based genome-scale modeling workflow^44^. Samples were stratified as M0, M1 or M2 macrophages, and age was collapsed into two groups: 2- and 6-month-old mice were assigned to the young group (m26), whereas 12- and 24-month-old mice were assigned to the aged group (m1224). Age-dependent metabolic remodeling was inferred using Compass 1.0 was coupled to the Gurobi Optimizer 13.0 mathematical programming solver to estimate sample-specific reaction activity scores under stoichiometric constraints^44,66^. The Mouse-GEM/Mouse1 was used as the reference genome-scale metabolic reconstruction, a curated Mus musculus metabolic model suitable for transcriptome-constrained simulations and multi-omics interpretation^45^.

Resulting matrices encoded metabolic features as rows and macrophage samples as columns. Analyses were performed independently for M1 and M2 macrophages to resolve polarization-specific effects of aging. Reaction metadata were generated by merging local Mouse-GEM metadata, official Mouse-GEM reactions.tsv records, and SBML-derived reaction names; unresolved entries were supplemented using curated reaction-name overlays and, where necessary, rule-based inference from reaction equations, subsystems, and cross-reference metadata. Metabolite annotations for uptake and secretion matrices were generated from official Mouse-GEM metabolite records and SBML species metadata, including names, formulas, charges, and compartment labels. Annotated outputs retained reaction name, equation, subsystem, gene association, compartment, and external identifiers where available.

For each polarization and metabolic matrix, young and aged groups were compared feature-wise. Group means, medians and standard deviations were computed, followed by the aged-minus-young difference in means, absolute mean difference, log2 fold-change and Cohen’s d effect size. Welch’s two-sample t-test was applied to accommodate unequal variances between age groups, and *P* values were adjusted using the Benjamini–Hochberg false-discovery-rate procedure. Features were ranked by absolute mean difference and then by nominal *P* value, prioritizing large and statistically supported shifts in inferred metabolic activity.

### Functional assays

Unlike previous studies using artificial fluoresbrite microspheres or bacteria, and a scratch assay to measure phagocytic activity and migration, respectively^32,50^, we used tumor cells as our target. BMDMs isolated from different age groups of UBC-GFP mice were seeded in a 96-well plate (13,000 cells/well) and polarized into M1- and M2-like phenotypes. After 24 hours of polarization, macrophages were co-cultured with SM1 (murine melanoma) cells stained with CellTrace Violet and pHRodo dyes. Cells were co-cultured for 12 hours in the ImageExpress Pico Imager (Molecular Devices) with images captured every 30 minutes. The percentage of phagocytosis was measured using CellReporterXpress software by quantifying the number of GFP macrophages positive for the pHRodo dye, which shows increased intensity at lower pH (4–9), as seen in phagosomes.

The changes in the antigen presentation machinery with aging were assessed by the ability of macrophages to present the SIINFEKL peptide. BMDMs from the four age groups were polarized to M1 phenotype with LPS and IFNγ for 24 hours. Cells were then treated with chicken ovalbumin (40 ng/mL) and harvested after four hours for flow cytometry. M0 macrophages and untreated M1 macrophages served as control samples. Cells were stained with Live/Dead fixable Aqua dead cell stain (Thermo Fisher), followed by antibodies against F4/80 (macrophage markers) and H2-kb-SIINFEKL. Flow cytometry analysis was performed to quantify H2-kb-SIINFEKL-positive cells. To determine the antigen cross-presentation ability of macrophages, we co-cultured M1 macrophages pulsed with ovalbumin (for four hours) with T-cells isolated from OT1 mice. Splenocytes were harvested from OT-1 mice, and T cells were purified using the Mouse T cell isolation kit (StemCell Technologies) according to the manufacturer’s protocol. T-cells were stained with Cell Trace Violet dye (ThermoFisher Scientific) at 1:4000 dilution and activated with Dyna Beads Mouse T-Activator CD3/CD28 (Gibco). Activated T-cells were seeded at a density of 40,000 cells/well in a 96-well U-bottom plate, along with 50 U/L of IL-2 (BioLegend). Macrophages were gently scraped using enzyme-free cell dissociation buffer and co-cultured with T-cells at 1:2 ratio (20,000 macrophages/well). After 72 hours of co-culture, cells were stained with CD8 antibodies and analyzed by flow cytometry. The decrease in Cell Trace Violet intensity determined the percentage of proliferating CD8 T cells.

In a 24-well trans-well system, BMDMs isolated from different age groups of UBC-GFP mice were seeded in the top insert. Macrophages were polarized to M1- and M2-like phenotypes and co-cultured with tumor cells in the bottom well. After 24 hours, images of the bottom well were captured with the ImageExpress Pico Imager (Molecular Devices) to quantify GFP-positive macrophages that migrated from the insert and to compare changes across age groups.

For individual gene expression changes associated with macrophage-specific functions, log2FC values relative to the age-matched “M0” group (e.g., 6mo M1 vs. 6mo M0) were used. Genes associated with GO-BP terms for “macrophage migration” (GO:1905517), “phagocytosis” (GO:0006909), and “antigen processing and presentation” (GO:0019882) were specifically investigated.

## Supplementary Material

Supplementary Files

This is a list of supplementary files associated with this preprint. Click to download.

- SureshetalnpjAgingSupplementaryInformation.docx
- SupplementaryFigure1.jpg
- SupplementaryFigure2.jpg
- SupplementaryFigure3.jpg
- SupplementaryFigure4.jpg
- SupplementaryFigure5.jpg
- image1.jpeg

## Data Availability

All data analyzed are represented as figures supporting the findings of this study and are available in this article and the supplementary file. The raw data generated from these figures are available from the corresponding authors upon reasonable request.

## CODE AVAILABILITY

The Python code and bioinformatic methods applied in differential expression analysis and FBA analysis are available from https://github.com/Nanocell-Lab/MacrophageAgingArticle.
